# Development of a robotic training curriculum for visceral and gastrointestinal surgical trainees: an international Delphi study

**DOI:** 10.1007/s00464-026-12781-x

**Published:** 2026-06-19

**Authors:** Michael G. Fadel, Josephine Walshaw, Marina Yiasemidou, Matthew Boal, Francesca Pecchini, Muhammed Elhadi, Lisa H. Massey, Francesco M. Carrano, Matyas Fehervari, Caoimhe M. Walsh, Piers R. Boshier, Jennifer Eckhoff, Peter Buckle, ERSC Study Group, Nicola de Angelis, Paolo Bianchi, Alexander Bloemendaal, Diego Cuccurullo, Sofia Esposito, Gianmaria Casoni Patticini, Micaela Piccoli, Mauro Podda, Elisa Reitano, María Rita Rodríguez Luna, Ernesto Tartaglia, Victor Tomulescu, Martin Wagner, Altaf Awan, Mark van Berge Henegouwen, Imran Bhatti, David Chan, Sonia Fernández-Ananin, Giovanni Maria Garbarino, Peter Grimminger, Benedetto Ielpo, Magnus Nilsson, Bijendra Patel, Alice Tsai, Christoph Tschuor, Gemma Vellalta, Ramon Villalonga, Manish Chand, Audrius Dulskas, Antonio D’Urso, Nicola Eardley, Eloy Espin-Basany, Giampaolo Formisano, Nikolaos Gouvas, Rosa Maria Jiménez-Rodríguez, Michail Klimovskij, Gianluca Pellino, Thalia Petropoulou, Wanda Petz, Kapil Sahnan, Theodosopoulos Theodosis, Ademola Adeyeye, Asma Afzal, Daniel Campioni-Norman, Bibek Das, Michael Devine, Lachlan Dick, Dolores Krauss, Gita Lingam, Pietro Mascagni, Tamsin Morrison, Munir Tarazi, Alexandra Aye Ivanuta, Peter A. Brennan, Michael Eichlseder, Samantha Evans, Neli Harvey, Ronalyn Hosena, Hannah Kettley-Linsell, Stella Mavroveli, Freideriki Sifaki, Tayana Soukup, Madeleine Wells, Lakshmi Balasubramanian, Niall Fahy, Harald Ginterstorfer, Elizabeth Jones, Sara Lazzaretti, David Marante, Gregory De Martino, Anders Melander, Shelley Petersen, James Scott, Mark Slack, Katherine Winger, Julie Hepburn, Pete Wheatstone, Suzanne S. Gisbertz, Nicole Bouvy, Alberto Arezzo, Silvana Perretta, Felix Nickel, Jim Khan, George B. Hanna, Barbara Seeliger, Stavros A. Antoniou, Hans F. Fuchs, Nader K. Francis, Christos Kontovounisios

**Affiliations:** 1 Department of Surgery and Cancer, Imperial College London, London, UK; 2 Leeds Institute of Medical Research, St James’s University Hospital, University of Leeds, Leeds, UK; 3 Department of Colorectal Surgery, The Royal London Hospital, Barts Health NHS Trust, London, UK; 4 The Griffin Institute, Northwick Park and St Mark’s Hospital, London, UK; 5 Division of General Surgery, Emergency and New Technologies, Baggiovara General Hospital, Modena, Italy; 6 Tripoli University Hospital, Tripoli, Libya; 7 College of Medicine, Korea University, Seoul, Republic of Korea (South Korea); 8 Department of Colorectal Surgery, Nottingham University Hospitals NHS Trust, Nottingham, UK; 9 Department of Medical and Surgical Sciences and Translational Medicine, Faculty of Medicine and Psychology, St Andrea Hospital, Sapienza University, Rome, Italy; 10 Department of Bariatric Surgery, Maidstone and Tunbridge Wells NHS Trust, Kent, UK; 11 Department of General, Visceral, Cancer and Transplantation Surgery, University Hospital Cologne, Cologne, Germany; 12 Department of Surgery, Amsterdam UMC location University of Amsterdam, Amsterdam, The Netherlands; 13 Cancer Center Amsterdam, Amsterdam, The Netherlands; 14 Department of Surgery, Leiden University Medical Centre, Leiden, The Netherlands; 15 Department of Surgical Sciences, University of Turin, Turin, Italy; 16 Institute of Image-Guided Surgery, IHU-Strasbourg, Strasbourg, France; 17 Department of Digestive and Endocrine Surgery, University Hospitals of Strasbourg, Strasbourg, France; 18 Department of General, Visceral and Thoracic Surgery, University Medical Center Hamburg-Eppendorf, Hamburg, Germany; 19 Department of Colorectal Surgery, Portsmouth Hospitals University NHS Trust, Portsmouth, UK; 20 ICube, UMR 7357, CNRS, INSERM U1328 RODIN, University of Strasbourg, Strasbourg, France; 21 Research Institute Against Digestive Cancer (IRCAD), Strasbourg, France; 22 Department of Surgery, Papageorgiou General Hospital, Thessaloniki, Greece; 23 2nd Surgical Department, HYGEIA Hospital, Athens, Greece; 24 Department of Surgery, NYU Grossman School of Medicine, New York, NY USA; 25 School of Medicine, National and Kapodistrian University of Athens, Athens, Greece; 26 Department of Colorectal Surgery, Chelsea and Westminster Hospital and Royal Marsden NHS Foundation Trust, London, UK

**Keywords:** Robotic surgery, Visceral surgery, Gastrointestinal surgery, Training, Education, Curriculum, Assessment, Certification

## Abstract

**Background:**

The rapid adoption of robotic surgical systems globally has created a critical gap in training, assessment and certification for visceral and gastrointestinal (GI) surgical trainees. This study, led by the European Association for Endoscopic Surgery (EAES), aimed to achieve an international consensus on a structured, platform-agnostic robotic training curriculum for GI surgical trainees.

**Methods:**

A 106-item Delphi questionnaire was developed with an international committee of surgical experts, trainees, methodologists and patient representatives. It was disseminated to a multidisciplinary panel of 83 GI robotic surgeons, trainees, human factors experts, robotic theatre team members and industry providers. Two Delphi survey rounds were conducted, with a priori consensus standard set at 70% or higher for agreement. A consensus meeting was subsequently held to discuss and finalise the items needed for a robotic training curriculum for GI surgical trainees.

**Results:**

Seventy-one (86%) participants from 15 countries completed round 1. A total of 82 items (77%) reached consensus and 32 new items were generated from free-text comments. Seventy of these participants (99%) completed the 56-item round 2 questionnaire, with 36 items (64%) reaching consensus and 5 new items generated. All 143 statements were discussed in the meeting and consensus was reached in the following areas: (i) key knowledge requirements of the bedside assistant and a console surgeon; (ii) training components; (iii) performance assessment and (iv) certification and supervision.

**Conclusion:**

International surgical experts, trainees and other key stakeholders reached consensus on the critical components of a platform-agnostic robotic training curriculum for GI surgical trainees. This will help shape the future of robotic surgical education and certification, promote standardised training practices and ultimately benefit patient safety and outcomes.

**Graphical abstract:**

**Figure:**
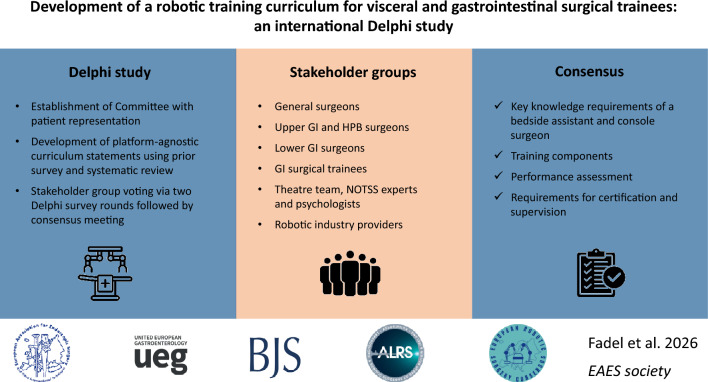

**Supplementary Information:**

The online version contains supplementary material available at 10.1007/s00464-026-12781-x.

Robotic surgery has rapidly expanded within the field of visceral and gastrointestinal (GI) surgery, which comprises disciplines including general, upper and lower GI, hepato-pancreato-biliary (HPB), endocrine, and bariatric surgery. The adoption of robotics has reshaped surgical practice, team dynamics, and the educational needs of trainees [1–3], driven by improvements in ergonomics, enhanced visualisation, and refined dexterity that facilitate complex minimally invasive procedures [4–6]. These advantages may potentially improve surgical quality and oncological outcomes for certain procedures [6–10]. Robotic systems are established in elective surgical practice globally, and are also progressively being introduced within the emergency setting [11–14].

However, the integration of robotic surgery has not been accompanied by the development of standardised surgical training programmes. Current approaches to robotic training remain variable across GI surgery, with a lack of standardisation in bedside-to-console progression, case-volume expectations, and assessment methods [2, 15]. In many hospitals, access to robotic platforms can be restricted by cost, system availability, or limited faculty expertise, who themselves required to complete their proficiency-gain curve. Furthermore, training often relies on industry-led programmes for specialities and sporadic courses and individual fellowships for residents rather than structured proficiency-based pathways and accreditation [16, 17]. As a result, GI surgical residents often report fragmented or insufficient exposure to robotic procedures, leading to differences in experience and progression to independent practice [18, 19].

A standardised, competency-based approach to robotic training in GI surgery is essential. Structured training curricula have been shown to enhance skill acquisition, facilitate progression along the learning curve, and provide a robust evidence base for credentialing and independent practice [20–22]. Despite growing international experience, there is currently no consensus amongst surgical speciality boards and professional societies on the training, assessment, and certification of GI trainees in robotic surgery.

To address these critical gaps, we established the European Robotic Surgery Consensus (ERSC) group to develop an evidence- and consensus-based curriculum for robotic training in GI surgery under the auspices of the European Association of Endoscopic Surgery (EAES) society in collaboration with its affiliated member, the United European Gastroenterology (UEG) society. Herein, we present the consensus process used to establish essential elements of a robotic curriculum for GI surgical trainees, including learning requisites, training components, assessment, and certification requirements to progress to independent and safe practice.

## Methods

### Project management and statement generation

**Fig. 1:**
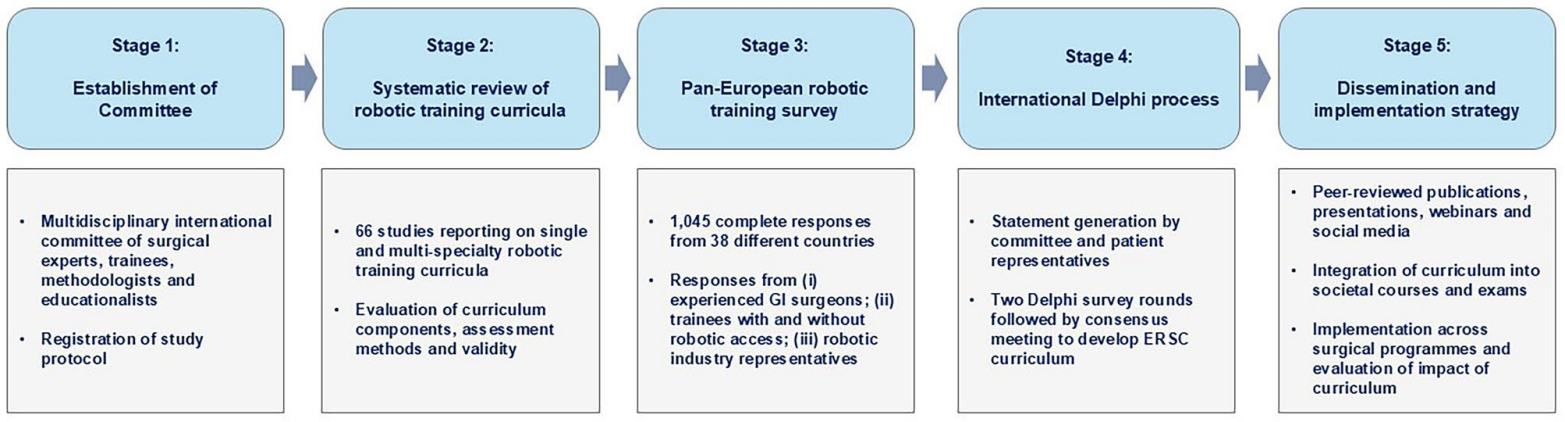
The five key stages for developing the ERSC robotic training curriculum for gastrointestinal and visceral surgery trainees. *ERSC* European Robotic Surgery Consensus; *GI* gastrointestinal.

An international project management committee (PMC) and an advisory executive committee (EC) established a study protocol outlining a structured, multi-stage consensus process [23] (Fig. 1). The PMC [MGF, JW, MY, MB, SAA, HFF, NFK, CK] was responsible for the day-to-day management and the EC [FP, ME, LHM, FMC, MF, SP, FN, JK, GBH, BS] provided strategic oversight of the project. The committee members are a multidisciplinary group of general, upper GI, HPB, and lower GI surgical experts and trainees, educational experts, and methodologists (Supplementary File 1).

Members of the PMC and EC held regular meetings to discuss and draft statements for the Delphi process using findings from our pan-European survey [17] and systematic review of multi-speciality robotic training curricula [20], as well as existing evidence [24–29]. The full search strategy of the systematic review is detailed in Walshaw et al. [20]. The drafted statements for the Delphi process were reviewed and finalised by the entire committee and two patient representatives. The statements were entered onto the Qualtrics XM software platform [30] and tested by the committee to check usability and technical functionality.

The first-round questionnaire contained 106 items, which were divided into four sections to help compose the GI robotic training curriculum: (i) knowledge requirements of the bedside assistant and console surgeon (43 items); (ii) training components (35 items); (iii) performance assessment (15 items) and (iv) requirements for certification and supervision (13 items).

### Participant selection

The appropriate selection of a group of experts with a high and concordant skill level is critical to promote robustness and data validity in Delphi studies [31, 32]. Key stakeholder groups for the Delphi panel were agreed upon by the committee and patient representatives. Participants were carefully selected on account of their expertise within these groups, along with their experience and interest in training and education: (i) independent, experienced GI surgeons (including general, HPB, upper and lower GI surgeons practising minimally invasive and/or robotic surgery); (ii) GI surgical trainees/residents; (iii) extended robotic theatre team including anaesthetists, scrub nurses and robotic practitioners; (iv) experts in technical skills competency assessment, non-technical skills for surgeons (NOTSS) experts and psychologists and (v) robotic industry representatives.

Based on observations from previous Delphi studies in the literature, we targeted a minimum total sample size of 60 expert participants to take part in the Delphi process [31, 33–36]. This sample size has been shown to have high replicability of results compared to those of larger sample sizes in Delphi studies [37]. Following the publication of the protocol [23], the decision was made to involve robotic industry representatives in the Delphi panel due to their experience in leading robotic training programmes and courses. Panel members were invited from European countries as well as global robotic industry providers. The committee recorded conflicts of interest and endeavoured to ensure a broad representation across the stakeholder groups, based on robotic experience and skills, demographics, and geographical location. We aimed to have representation from at least ten European countries and a minimum of six representatives within each stakeholder group. To ensure equal opportunities for each industry company, we aimed for a minimum of four industry providers with up to three representatives from the same company participating in the voting process. This was to mitigate any potential conflicts and reinforce the platform-agnostic nature of the curriculum, particularly given the current number of robotic platforms emerging on the market.

### Delphi survey rounds

The Delphi methodology is a structured, iterative process to acquire knowledge and opinions from experts on a selected topic [38, 39]. It has been widely adopted for establishing guidelines and recommendations in the medical and surgical field [40–42]. Whilst a virtual Delphi approach provides participant anonymity, which may allow for more open expression of views [43, 44], it also has the potential disadvantage of lacking group interaction for consensus building [33]. For this reason, we arranged two online questionnaire rounds followed by a hybrid consensus meeting enabling members to provide further clarification on specific issues and present arguments to justify their viewpoints.

The two rounds of the Delphi survey were conducted using the Qualtrics XM software, with a demographics questionnaire included in round 1. Participants were emailed a website link prompting them to complete the Delphi survey. Members of the Delphi panel provided their responses to each item using a Likert scale following the Grading of Recommendations Assessment, Development and Evaluations (GRADE) 9-point scale [34–36, 45]: (i) 1–3 = of limited importance (item should not be included in the robotic training curriculum); (ii) 4–6 = important but not critical (item should be discussed in the consensus meeting) and (iii) 7–9 = important and critical (item should be included in the robotic training curriculum). A ‘do not know’ option was included for participants who did not feel qualified to rank any specific item. The panel had the opportunity to suggest additional statements or modifications to the statements in free-text fields.

Each round was open for approximately 4 weeks. Responses were analysed and summarised by the PMC between each round, and the following consensus definitions [36, 45] were used: (i) consensus for inclusion: ≥ 70% of participants scoring 7–9 and < 15% of participants scoring 1–3; (ii) consensus for exclusion: ≥ 70% of participants scoring 1–3 and < 15% of participants scoring 7–9 and (iii) no consensus for inclusion or exclusion: failure to achieve either of the above.

### Consensus meeting

The overall aim of the hybrid consensus meeting was to discuss and agree on the final statements needed for a robotic training curriculum for GI trainees. The meeting was held over one full day at the Hammersmith Hospital Campus, Imperial College London (London, United Kingdom). Committee members and stakeholder group representatives were invited to participate in the consensus meeting, with a maximum target of 30 participants in total, as larger meetings can limit interaction amongst participants [46]. The committee selected participants for the meeting from those who registered their interest based on (i) their ability to attend the meeting on proposed dates and times and (ii) the need to have an international multidisciplinary group of stakeholders.

Prior to the consensus meeting, all participants were sent the meeting agenda, participant list, summary of the survey and systematic review findings, and the results of the Delphi survey rounds via email. The consensus meeting itself intended to (i) ratify inclusion of all items that reached consensus in the Delphi survey; (ii) discuss and anonymously vote (on Qualtrics XM) for inclusion (≥ 70% voting to include or exclude) of items that had not reached consensus and (iii) discuss merging and wording of all items in the final curriculum [45–47]. The meeting was facilitated by an independent chair (P.B., a Professor in Human Factors) and a co-chair (M.G.F., a GI surgical trainee). The chair provided a structured interaction amongst participants to ensure all members had equal opportunities to contribute throughout the meeting. The importance of developing an international, feasible, platform-agnostic curriculum that included only critical statements was emphasised to participants during the meeting. Items that were platform-specific but considered critical were discussed for inclusion. Discussion of items during the meeting was audio recorded (with participant verbal consent) to facilitate comprehensive minutes and maintain a record of decisions made. Dissemination and implementation strategies, along with future evaluation of developed outputs, were also discussed. Following the Delphi process, the curriculum phases and key components were finalised by the committee and members of the study group.

### Statistical analysis

Only fully completed responses from rounds one and two of the survey were included in the analysis. Descriptive statistics were applied to summarise participant demographics and responses across each round. The percentage of participants that rated an item with a score of 1–9 using the GRADE 9-point scale was calculated. All analyses were performed using IBM SPSS Statistics, Version 28.0 (Chicago, USA).

## Results

### Demographics of participants

Eighty-three participants were invited to take part in round 1 of the Delphi process, with 71 participants (86%) completing all components (Table 1). Respondents were from 15 countries, with the top six contributing countries being the UK (*n* = 25, 35%), Italy (*n* = 16, 23%), Spain (*n* = 8, 11%), the Netherlands (*n* = 4, 6%), Germany (*n* = 3, 4%), and Greece (*n* = 3, 4%). Five industry providers participated including Asensus Surgical (Durham, NC, USA), CMR Surgical (Cambridge, UK), Intuitive Surgical (Sunnyvale, CA, USA), Medtronic (Minneapolis, MN, USA) and Surgical Science (Gothenburg, Sweden). The ERSC study group and members of the Delphi panel are shown in Supplementary File 2. A total of 28 participants attended the consensus meeting. This comprised eight committee members, who were all experienced GI surgeons, and 20 stakeholders from the ERSC study group, including experienced GI surgeons (n = 3), trainees (*n* = 6), theatre team staff (*n* = 4), NOTSS experts and psychologists (*n* = 2), robotic industry providers (n = 4) and a patient representative.

**Table 1:** Participant demographics that completed round one of the Delphi process

Characteristics	Participants, *n* = 71 (%)
Age	
21–30	1 (1.4%)
31–40	22 (31%)
41–50	30 (42%)
51–60	12 (17%)
≥ 61	6 (9%)
Sex	
Male	43 (61%)
Female	28 (39%)
Countries (*n* = 15)	
Austria	1 (1.4%)
Cyprus	1 (1.4%)
Denmark	1 (1.4%)
France	2 (3%)
Germany	3 (4%)
Greece	3 (4%)
Ireland	1 (1.4%)
Italy	16 (23%)
Lithuania	1 (1.4%)
Netherlands	4 (6%)
Romania	1 (1.4%)
Spain	8 (11%)
Sweden	2 (3%)
UK	25 (35%)
USA	2 (3%)
Stakeholder groups	
General surgeons	16 (23%)
Upper GI & HPB surgeons	15 (21%)
Lower GI surgeons	15 (21%)
GI surgical trainees/residents	10 (14%)
Theatre team members, NOTSS experts and psychologists	7 (10%)
Robotic industry representatives	8 (11%)
Surgical sub-specialty	
Bariatric	2 (3%)
Emergency & trauma	1 (1.4%)
General	16 (23%)
HPB	4 (6%)
Hernia/abdominal wall	2 (3%)
Lower GI	20 (28%)
Surgical oncology	1 (1.4%)
Upper GI	12 (17%)
Other	5 (7%)
Hospital type	
Public university (teaching)	53 (75%)
Public non-university (non-teaching)	3 (4%)
Private	3 (4%)
Other	4 (6%)
Not applicable	8 (11%)
Robotic platform	
Da Vinci	54 (76%)
Hugo	4 (6%)
Versius	4 (6%)
Senhance	1 (1.4%)
Other	3 (4%)
Surgeon operative experience	
Bedside assisted more than 50 cases	33 (72%)
Performed more than 50 cases independently	31 (67%)
Performed more than 200 cases independently	19 (41%)

*NOTSS* Non-Technical Skills for Surgeons; *GI* gastrointestinal; *HPB* hepato-pancreato-biliary

### Overview of Delphi process

**Fig. 2:**
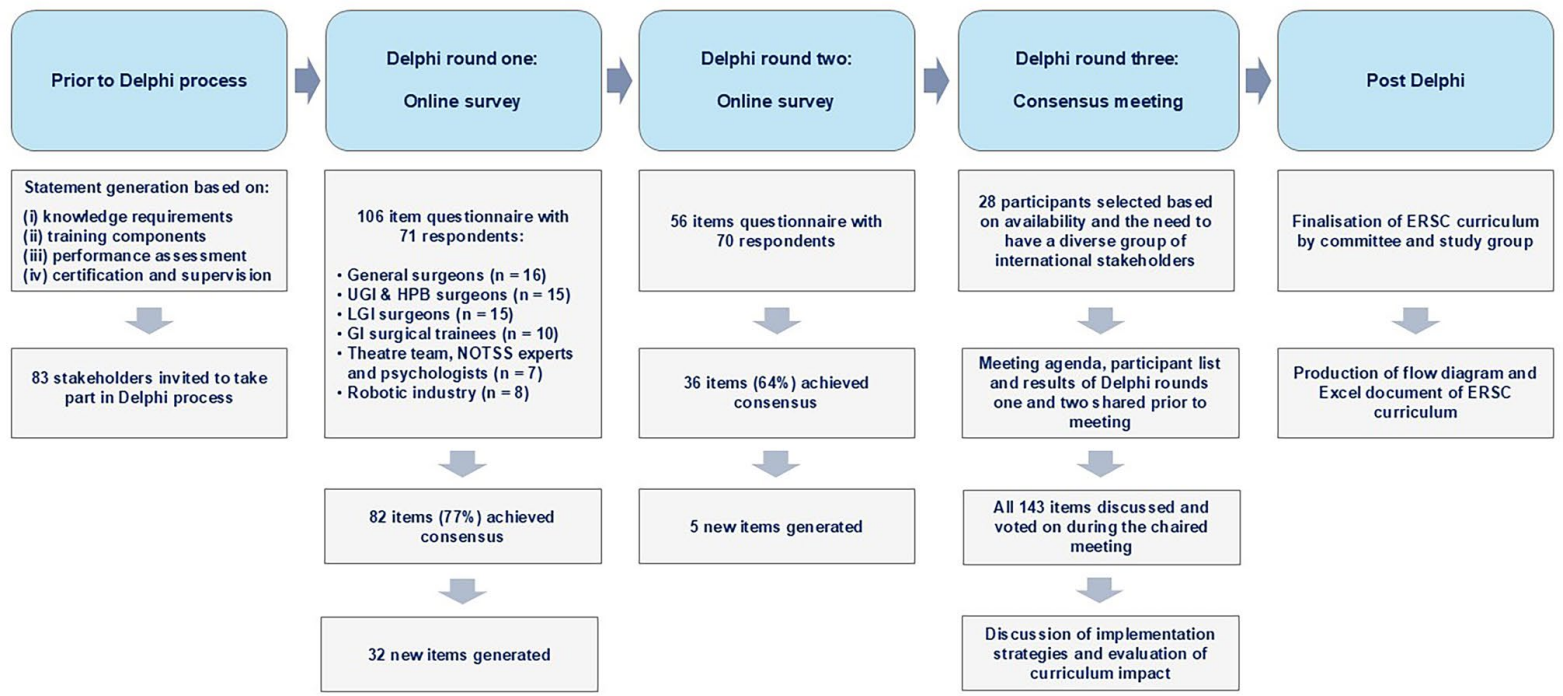
Delphi methodology to reach consensus on the fundamentals of the ERSC robotic training curriculum for gastrointestinal and visceral surgical trainees. *ERSC* European Robotic Surgery Consensus; *HPB* hepato-pancreato-biliary; *NOTSS* non-technical skills for surgeons; *LGI* lower gastrointestinal; *UGI* upper gastrointestinal

In round 1, 82 items (77%) from 106 items reached consensus, with 32 new items generated (Fig. 2). Seventy of the 71 respondents (99%) subsequently completed all components of the second round. In this round, 56 items (24 items that did not reach consensus and 32 new items in round 1) were presented, with 36 items (64%) achieving consensus and five new statements generated. All 143 items (106 initial items and 37 new items from rounds 1 and 2) were discussed and voted on during the consensus meeting. Following the Delphi process, the committee and panel finalised the curriculum components, comprising 57 statements spanning key domains based on knowledge requirements, training components, performance assessment and certification and supervision standards.

### Knowledge requirements of the bedside assistant and console surgeon trainee

Delphi panellists agreed that preoperative knowledge of the theatre room set-up and patient positioning (94%, round 1), and draping the robotic system (70%, round 2) were essential core skills for both the bedside assistant and console surgeon trainee (Table 2). The intraoperative key skills of a bedside assistant and console surgeon should include safe entry, docking, troubleshooting, inserting and changing robotic instruments, tissue handling, emergency undocking, and conversion. Additional core skills were identified for the console surgeon, including demonstrating control and use of the foot pedals and hand clutches (99%, round 1), multi-arm control of the robotic instruments (96%, round 1) and tissue dissection (97%, round 1). Demonstrating suture handling and knot tying was also deemed critical for the console surgeon (97%, round 1). Non-technical skills, such as situational awareness and team communication, were deemed essential for both the bedside assistant and console surgeon trainee, with leadership skills (96%, round 1), ergonomic awareness (80%, round 2) and time management (80%, round 2) believed to be specific skills essential for the console surgeon. The voting scores of the items that reached consensus, as well as those that did not, are shown in Supplementary File 3.

**Table 2:** Curriculum knowledge requirements for a bedside assistant and console surgeon trainee with consensus scores of critical importance (7–9) in round one and two

Item	Section 1: Knowledge requirements of the bedside assistant and console surgeon	Round one (score 7–9)	Round two (score 7–9)
Key skills of a bedside assistant and console surgeon should include			
	*Preoperative*		
1	Knowledge of the theatre room set-up and patient position	94%	-
2	Knowledge of the console and its functions	97%	-
3	Knowledge of how to drape the robotic system	69%	70%
4	Knowledge of the physiology of pneumoperitoneum	89%	-
	*Intraoperative*		
5	Be able to perform safe entry of trocars, port placement and closure	93%	-
6	Be able to dock the robotic system safely	90%	-
7	Be able to troubleshoot and re-dock the robotic system	90%	-
8	Be able to insert, change and remove robotic instruments	90%	-
9	Be able to appropriately use assistant port and maintain a clear image	85%	-
10	Be able to demonstrate correct techniques for tissue handling	97%	-
11	Be able to manage intraoperative complications (e.g. bleeding)	97%	-
12	Be able to convert to laparoscopic/open surgery	97%	-
13	Be able to perform planned and emergency undocking	97%	-
14	Be able to manage collisions/arm clashes	-	96%
	*Non-technical skills*		
15	Be able to demonstrate situational awareness	99%	-
16	Be able to demonstrate effective communication skills with the team	99%	-
Additional key skills of a console surgeon should include:			
	*Preoperative*		
17	Knowledge of procedure-specific port placement	97%	-
	*Intraoperative*		
18	Be able to demonstrate master manipulator control use of the foot pedal and hand clutches (including camera control)	99%	-
19	Be able to demonstrate multi-arm control of the robotic instruments	96%	-
20	Be able to demonstrate correct techniques for tissue dissection	97%	-
21	Be able to demonstrate suture handling and knot tying	97%	-
	*Non-technical skills*		
22	Be able to demonstrate leadership skills	96%	-
23	Be able to demonstrate ergonomic awareness and fatigue management	-	80%
24	Be able to demonstrate time management and pacing	-	80%

### Training components

E-learning modules were considered critical (82%, round 1) in providing theoretical knowledge on robotic consoles set-up and troubleshooting (100%, round 1), patient selection and preparation (92%, round 1), port placement (97%, round 1) and docking (99%, round 1) (Table 3). Demonstrations of common robotic procedures (e.g. gastrectomy, hemicolectomy), emergency management, and non-technical skills were also deemed essential modules. Non-technical skills may also be covered during e-learning through theoretical knowledge learning (e.g. types of leadership styles, safety checklists), case studies and videos of simulated scenarios, followed by reflective learning exercises. It was agreed that there should be a pass/fail knowledge assessment at the end of the e-learning, and that, over time, robotic curricula should be detailed and integrated into these modules. Core skills/device training on port positioning, docking, emergency conversion, and non-technical skills, through team-based scenarios, was deemed critical (97%, round 1), along with proficiency-based, rather than time-based, virtual reality simulation (83%, round 1).

**Table 3:** Curriculum components of the gastrointestinal surgery robotic training programme with consensus scores of critical importance (7–9) in round one and two

Item	Section 2: Training components	Round one (score 7–9)	Round two (score 7–9)
Key components of a robotic training curriculum for GI surgical trainees should include			
25	E-learning modules	82%	-
26	Core skills/device training (patient and port positioning, docking, non-technical skills, emergency undocking and conversion)	97%	-
27	Virtual reality simulation training (proficiency-based training including endowrist manipulation, camera control and tissue dissection)	83%	-
28	Dual console training (where applicable)	83%	-
29	Dry-lab simulation (high-fidelity models), e.g. familiarisation with robotic instruments, use of energy and haemostasis, advanced dissection skills	96%	
30	Wet-lab simulation (animal or cadaveric)	66%	70%
31	Bedside assisting	89%	-
32	Live case observation (observation of robotic operations performed by an expert robotic surgeon)	94%	-
Robotic e-learning modules for GI surgical trainees should include			
33	Introduction to robotic systems and principles of ergonomics	-	93%
34	How to set up robotic console and troubleshooting	100%	-
35	Patient selection and preparation	92%	-
36	Port placement	97%	-
37	Docking	99%	-
38	Demonstration of common procedures	-	93%
39	Emergency management/conversion to open or laparoscopy	96%	-
40	Knowledge assessment	75%	-
Dry- and wet-lab robotic simulation metrics for GI surgical trainees should capture			
41	Completion of tasks	90%	-
42	Task scores	96%	-
43	Error rate (e.g. instrument clash, loss of instrument view)	87%	-
44	Proficient camera control	90%	-
45	Appropriate use of the third arm	83%	-
46	Use of hand and foot controllers at the console	94%	-
47	Economy of movement in tissue dissection and suturing	86%	-
48	Safe energy application	93%	-
49	Hand-eye coordination efficiency	-	90%

*GI* gastrointestinal

Dry-lab simulation, ideally using high-fidelity 3D anatomical models (96%, round 1), if available, is essential for becoming familiar with robotic instruments and energy use for enhancing advanced dissection skills. Wet-lab simulation, with either animal or cadaveric models, was introduced into the curriculum to develop real tissue handling skills (70%, round 2). Wet-lab simulation, with either animal or cadaveric models, was introduced into the curriculum to develop real tissue handling skills; however, it was acknowledged that trainees may not necessarily be exposed to this due to legal restrictions in their country and other ethical and practical considerations, such as cost and availability. Dry and wet-lab simulation metrics deemed critical included completion of tasks (90%, round 1) with associated scores (96%, round 1), error rate (87%, round 1), camera control (90%, round 1), economy of movement in tissue dissection and suturing (86%, round 1), and safe energy application (93%, round 1). Time spent on a given task (58%, round 1 and 57%, round 2) and the number of attempts (63%, round 1 and 61%, round 2) were believed to be less important. These metrics represent domains of performance captured by robotic simulators rather than prespecified competency thresholds, which should be defined by validated simulator benchmarks. Although currently platform-specific, dual console training was deemed important for trainee learning and mentoring (83%, round 1). However, it was also acknowledged that access to dual consoles for trainees, whether on site or remotely in a telesurgery set-up, may be limited by institutional costs and theatre space requirements. Alongside simulation training, trainees should be able to progress to bedside assisting (89%, round 1) and live case observation (94%, round 1).

### Performance assessment

When operating on the console, GI surgical trainees should capture the number of cases performed for each procedure (83%, round 1) and routine clinical outcome data (89%, round 1), such as intraoperative [48] or postoperative [49] complications, unplanned conversion rate and return to theatre (Table 4). Recording the number of hours operating on the console and the number of key steps of the procedure completed was deemed less critical. The technical skills of GI trainees should be assessed using a combination of case logs (73%, round 1), global rating scales, e.g. GEARS [50, 51] (80%, round 1), procedure-specific tools, e.g. Competency Assessment Tools (CATs) [52] (83%, round 1) and video assessment (87%, round 1). In terms of video assessment, the Delphi panel agreed that the key steps of an operative video should be assessed (73%, round 2), rather than the full length of the video, to ensure feasibility. The video assessment should ideally be performed by a minimum of two experts including the proctor and a blinded expert (73%, round 2). Another blinded expert may be required if there is significant disagreement between the two initial experts.

**Table 4:** Performance assessment, certification and supervision requirements of the gastrointestinal surgery robotic training curriculum with consensus scores of critical importance (7–9) in round one and two

Item	Section 3: Performance assessment	Round one (score 7–9)	Round two (score 7–9)
When robotic console operating, clinical metrics for GI surgical trainees should capture			
50	Number of procedures performed	83%	-
51	Routine clinical outcome data recorded for cases e.g. intra- and postoperative complication rates, return to theatre, readmissions, unplanned conversion rate to open or laparoscopy, histopathological outcomes (if applicable)	89%	-
Technical skills assessment for GI surgical trainees to progress to independent practice should include			
52	Case logs	73%	-
53	Validated objective assessments (e.g. GEARS)	80%	-
54	Procedure-specific objective assessment tools (e.g. CAT)	83%	-
55	Video assessment using objective tools—key steps of operative video assessed by a minimum of two experts (e.g. by proctor and a blinded expert)	87%	-

Item	Section 4: Recommended requirements for certification and supervision		
The following are required for robotic platform sign-off for GI surgical trainees			
56	Completion of robotic procedures independently—trainer/proctor should deem the trainee as being able to practice independently and safely (e.g. through the use of objective assessment metrics/video assessment and clinical outcome metrics)	93%	-
57	Stepwise progression on acquiring skills and demonstrate this in cases of increasing complexity	85%	-

*CAT* Competency assessment tool, *GEARS* Global evaluative assessment of robotic skills, *GI* gastrointestinal

### Requirements for certification and supervision

The Delphi panel reached consensus that a trainee should be primarily signed off as practically safe and independent by the trainer/proctor (93%, round 1). Trainees should demonstrate stepwise progression in acquiring skills during this training process and do so in cases of increasing complexity (85%, round 1). The completion of a robotic fellowship programme was deemed desirable but not mandatory in this curriculum (63%, round 1 and 66%, round 2), especially due to a lack of available robotic fellowships even at an international level.

**Fig. 3:**
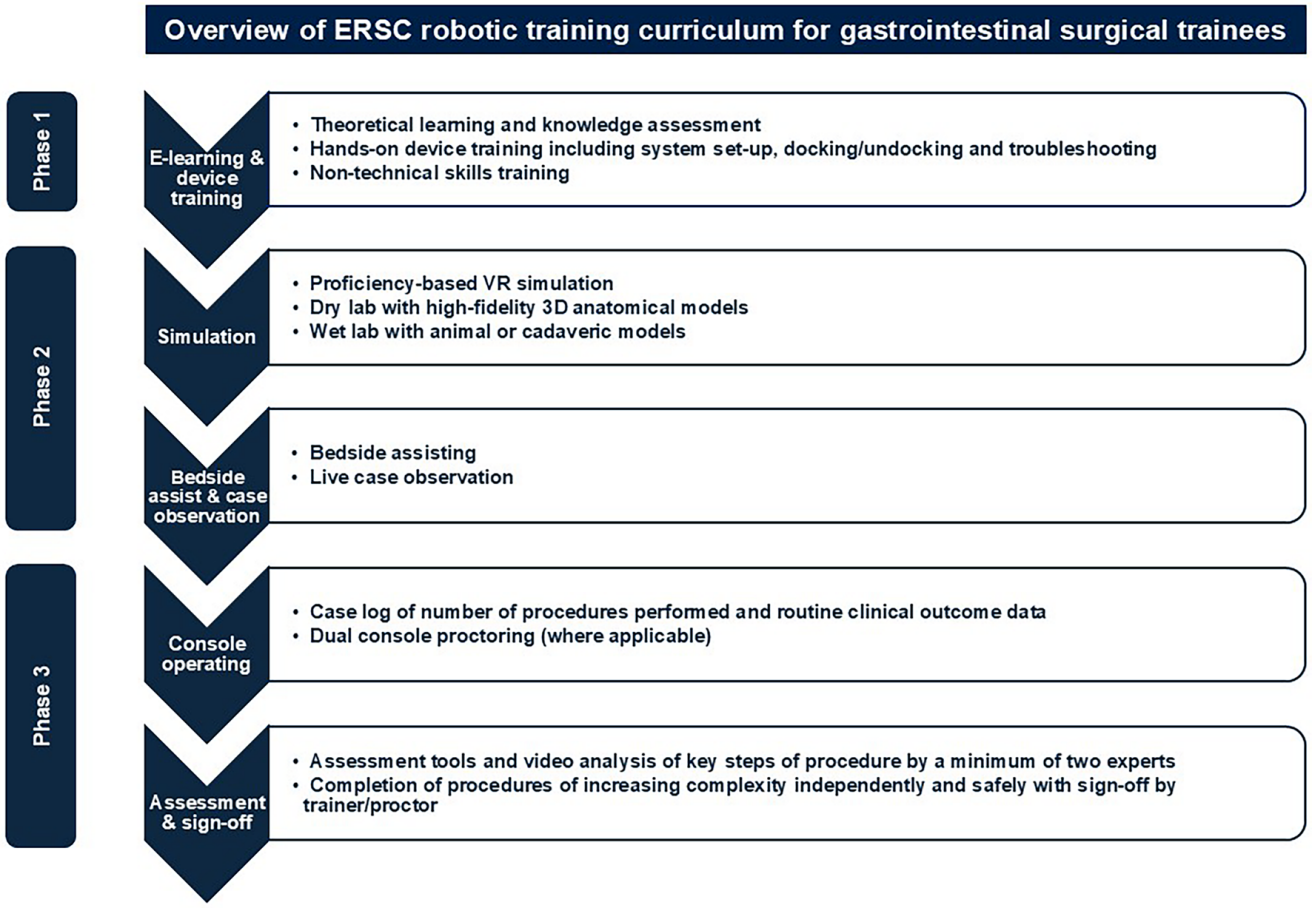
Overview of the key phases and steps of the proposed ERSC robotic training curriculum required for gastrointestinal and visceral surgical trainees. *ERSC* European Robotic Surgery Consensus; *VR* virtual reality

The long-term impact of the robotic training curriculum can be primarily assessed by the number of procedures performed per year by a given trainee (80%, round 1) and their related clinical outcomes (e.g. complication rates per year) (86%, round 1). Overall, the curriculum should lead to a trainee demonstrating a gradual reduction in the percentage of assisted cases, coinciding with an increase in the percentage of performed and trained cases in cases of growing complexity. A flow diagram (Fig. 3) and a copy of the phased ERSC robotic training curriculum is available on Microsoft Excel (Supplementary File 4).

## Discussion

This Delphi study proposes an evidence-based, comprehensive international framework for a platform-agnostic robotic training curriculum for GI surgical trainees, ultimately aiming to produce robotic surgeons who can practise independently and safely. The proposed ERSC curriculum is unique as it spans all stages of training, from novice to bedside assistant and console surgeon, and encompasses both technical and non-technical skills. Training is delivered through a structured approach, incorporating e-learning for theoretical understanding; simulation (virtual, dry, and wet labs) for hands-on practice; bedside assisting and live case observation; and supervised console operation until expert-assessed sign-off and certification. There should be a shift from the subjective assessment of trainees to the use of standardised, validated objective assessment tools to measure surgical performance. Certification can be undertaken by a qualified proctor if the trainee demonstrates safe and independent operation.

The Delphi panel agreed on core competencies for the bedside assistant and the console surgeon, reflecting the complementary roles in robotic procedures, with additional competencies needed for console surgeons. For example, bedside assistant trainees must be proficient in robot set-up, safe docking, instrument exchange, troubleshooting, and emergency undocking, ensuring patient safety and efficient teamwork. Console surgeons are expected to also master advanced technical skills including multi-arm instrument control, precise tissue handling and dissection, alongside non-technical skills such as leadership and time management. The Delphi process affirmed that these role-specific competencies should be explicitly addressed in training.

Panellists strongly supported didactic e-learning modules on practical topics to ground trainees in the fundamentals of robotics. There was also consensus on the need for simulation training as a progressive preparatory step before bedside assisting and console operating, aligning with prior structured curricula [22], such as the Fundamentals of Robotic Surgery curriculum [53] and the European Urology Programme [54], both of which emphasise proficiency-based progression. The Delphi panel agreed on important dry-lab and wet-lab simulation metrics, however, it should be acknowledged that further work is required to define these metrics and ensure they provide discriminatory value in training. Dual console training was highlighted by the Delphi panel as an important enabler of effective real-time training [55–57]. It should be taken into account that dual console training is currently platform-specific and can be limited by theatre space and associated costs, and is therefore unlikely to be universally accessible for trainees across Europe.

A major focus of the consensus was establishing objective performance and clinical metrics on patient outcomes for assessment and certification. The panel agreed that trainee progression should be evaluated using a combination of global objective assessment tools and procedure-specific metrics. Summative assessments at the end of a defined interval were deemed more practical and feasible, rather than continuous assessments during training. Structured rating scales, such as GEARS [50] and procedure-specific CATs [52, 58], were endorsed to provide measures of technical proficiency, complemented by the monitoring of clinical metrics, including complications and unplanned conversion rates. This mirrors the competency-based credentialing models advocated in other specialties, where a combination of simulation benchmarks and intraoperative performance data is used to sign-off trainees [54].

The panel did not reach a consensus on routine full-length video assessments by external raters, reflecting an ongoing debate about the feasibility of examiner agreement in video-based evaluations. Instead, a more focused approach assessing key video segments of an operation with input from both a blinded expert reviewer and the trainee’s proctor was considered more practical and feasible [59, 60]. It has also been shown there is minimal difference in operative assessment when raters used shorter, condensed videos compared to full-length videos [61]. Expert consensus of procedures [61, 62] can be used to define the critical steps of a procedure for video assessment. The adoption of artificial intelligence (AI)-based video assessment may also be considered in the future, once concerns regarding technical robustness, safety, and ethical challenges have been addressed. For example, EndoDigest, an AI platform, has recently been developed and validated to trim full-length videos down to key steps to reliably speed up video-based assessments [63, 64].

Validity and reliability considerations underpinning this Delphi process merit discussion. Content validity was built by deriving statements from a prior systematic review [20] and stakeholder survey work [17], ensuring grounding in evidence and real-world training gaps. The high consensus rates (consensus agreed on 77% of items in round 1 and 64% of remaining items in round 2) suggest face validity amongst a broad group of key stakeholders and experts. However, institutional and legal differences across regions may limit transferability. Training requirements and credentialing vary internationally; some countries or hospitals mandate formal certification or a minimum number of cases, whilst others rely on less structured sign-offs. The consensus aims to reflect a primarily European context and platform-agnostic curriculum, assuming the feasibility of elements such as wet-lab training, standardised assessments, and dedicated proctoring, which may not be uniformly available. Thus, whilst the consensus provides a strong template, local adaptation will be necessary.

### Future work

For further development of the proposed ERSC curriculum, we are following Kern’s framework to implement and evaluate the effectiveness of the curriculum. The curriculum will be integrated within societal courses, international exams and surgical training programmes, following the Laparoscopic Colorectal Surgery Training Programme (LAPCO) model [21], in collaboration with surgical societies, such as EAES and UEG, and industry. Competency level scores will be developed and added to the curriculum, and knowledge and skills acquisition of participants will be assessed using pre- and post-curriculum performance. Long-term participant performance after curriculum completion can be tracked using operative case numbers and outcomes over time. Feedback obtained from learners and trainers will also allow for further fine-tuning and pedagogical alignment of the curriculum, and the curriculum can be updated as necessary.

The successful implementation of the ERSC curriculum will require coordinated efforts from training institutions, professional bodies, and industry partners. The curriculum’s modular nature will lend itself to flexible adoption; training centres could integrate the e-learning component into existing didactic teaching, utilise available simulators for the recommended dry and virtual lab sessions, and collaborate with regional labs or industry courses to provide a wet-lab experience. Incorporating bedside assisting and, where feasible, dual console cases as important components will require scheduling trainees in those roles, which may impact operative workflow. Institutions could address this by establishing formal robotic mentorship programmes where an experienced robotic surgeon (or a team of mentors) is accountable for guiding trainees through the bedside-to-console progression. Train-the-trainer programmes may be necessary to familiarise mentors with curriculum expectations and assessment tools, ensuring evaluations remain calibrated and fair across different sites [65]. Moreover, incorporating the curriculum into existing surgical training timelines will necessitate acknowledging that robotic training is now an integral part of GI surgery education, warranting dedicated time and resources. Future implementation should involve the wider multidisciplinary team, with surgical assistants, scrub nurses and anaesthetists included in training to strengthen teamwork, safety and efficiency.

### Strengths and limitations

Despite the robust methodological process adopted, several limitations must be acknowledged. A relatively large proportion of the Delphi panel was from the UK (35%), Italy (23%) and Spain (11%), with limited representation from Eastern and Northern Europe. This could reduce the generalisability to settings with different case volumes, infrastructure, or access to robotic platforms. Limited participation from low- and middle-income countries may mean that perspectives on implementing training in resource-limited settings have been underrepresented. In addition, there is potential response and selection bias, as the panel largely consisted of early adopters and robotic enthusiasts, many of whom had performed more than 50 independent robotic cases. This may have introduced bias towards more intensive training requirements and optimistic assumptions about resource availability. We attempted to mitigate for this by including a range of stakeholder roles and by allowing open discussion and justification during the consensus meeting. Furthermore, Delphi studies often rely purely on expert opinion to generate findings and in some cases specific statements may be best addressed through other approaches, such as a systematic review of research evidence.

We have set out to develop a robotic training curriculum for GI surgical trainees broadly, without stratification by subspeciality. This broad applicability is a strength but may obscure nuanced subspeciality differences. For example, the new European coloproctology guideline for robotic training emphasises steps specific to colorectal resections, such as total mesorectal excision, as key competencies for certification [27]. We present a foundational framework that can be augmented by speciality or national boards with procedure-specific requirements as needed. Overall, the consensus aligns with the direction of recent multi-speciality frameworks, such as the ORSI Consensus Meeting on European Robotic Training [66], which identified common training needs, whilst adding curriculum content specific to GI surgery. In particular, the panel emphasised that non-technical skills training, team-based aspects, and stepwise certification requirements add an important dimension to curricula, complementing the technical skills focus of earlier programmes.

## Conclusion

This Delphi-based consensus provides the framework for an international, platform-agnostic curriculum for GI surgical trainees in robotic surgery. It specifies the skills to be learned, the methods of acquisition, and the approaches to competency assessment and certification, providing a framework to standardise training and improve the quality of robotic surgical education. A rigorous, evidence-informed consensus process supports the validity of our recommendations. By addressing both technical and non-technical competencies, and outlining a structured yet flexible learning pathway, the proposed curriculum components can help synchronise robotic training across diverse training programmes and can be further adapted for dedicated subspecialty training. Future efforts should focus on piloting this curriculum framework in real-world settings, evaluating its impact on trainee performance and confidence, and patient outcomes, with further refinement based on those insights. With continual updates as technology and techniques evolve, and potential expansion to other regions, the curriculum can serve as a foundation for credentialing models, ensuring that the next generation of GI surgeons is safely and proficiently prepared to operate with robotic technology, ultimately improving patient outcomes.

## Supplementary Information

Below is the link to the electronic supplementary material.

Supplementary file1 (DOCX 50 KB)

Supplementary file1 (XLSX 16 KB)
