# Supplementary material for: Development of a robotic training curriculum for visceral and gastrointestinal surgical trainees: an international Delphi study
Source: Surg Endosc. 2026 Jun 19;40(8):6913–27. doi: 10.1007/s00464-026-12781-x (PMC13457438; doi:10.1007/s00464-026-12781-x)
Supplement: Supplementary file 1 [file 464_2026_12781_MOESM1_ESM.docx]

**Supplementary file 1.** Project Management and Advisory Executive Committee members. GI, gastrointestinal; HPB, hepato-pancreato-biliary.

| **Committee members** | **Role** | **Country** |
| --- | --- | --- |
| Stavros Antoniou | General surgeon | Greece |
| Matthew Boal | Surgical trainee | United Kingdom |
| Francesco Maria Carrano | General surgeon | Italy |
| Muhammed Elhadi | Surgical trainee | Libya |
| Michael Fadel | Surgical trainee | United Kingdom |
| Matyas Fehervari | Upper GI surgeon | United Kingdom |
| Nader Francis | Lower GI surgeon | United Kingdom |
| Hans Fuchs | Upper GI surgeon | Germany |
| George Hanna | Upper GI surgeon | United Kingdom |
| Jim Khan | Lower GI surgeon | United Kingdom |
| Christos Kontovounisios | Lower GI surgeon | Greece |
| Lisa Massey | Lower GI surgeon | United Kingdom |
| Felix Nickel | HPB surgeon | Germany |
| Francesca Pecchini | Surgical trainee | Italy |
| Silvana Perretta | Upper GI surgeon | France |
| Barbara Seeliger | General surgeon | France |
| Josephine Walshaw | Surgical trainee | United Kingdom |
| Marina Yiasemidou | Lower GI surgeon | United Kingdom |

**Supplementary file 2.** Study group members.

| **Role** | **Study group member** | **Country/provider** |
| --- | --- | --- |
| **General surgeons** | Nicola de Angelis | Italy |
|  | Alberto Arezzo | Italy |
|  | Paolo Bianchi | Italy |
|  | Alexander Bloemendaal | Netherlands |
|  | Nicole Bouvy | Netherlands |
|  | Diego Cuccurullo | Italy |
|  | Sofia Esposito | Italy |
|  | Gianmaria Casoni Pattacini | Italy |
|  | Micaela Piccoli | Italy |
|  | Mauro Podda | Italy |
|  | Elisa Reitano | France |
|  | María Rita Rodríguez Luna | France |
|  | Ernesto Tartaglia | Italy |
|  | Vincenzo Trapani | Italy |
|  | Victor Tomulescu | Romania |
|  | Martin Wagner | Germany |
| **Upper GI/HPB surgeons** | Altaf Awan | United Kingdom |
|  | Mark van Berge Henegouwen | Netherlands |
|  | Imran Bhatti | United Kingdom |
|  | Piers Boshier | United Kingdom |
|  | David Chan | United Kingdom |
|  | Sonia Fernández-Ananin | Spain |
|  | Giovanni Maria Garbarino | Italy |
|  | Suzanne Gisbertz | Netherlands |
|  | Peter Grimminger | Netherlands |
|  | Benedetto Ielpo | Spain |
|  | Magnus Nilsson | Sweden |
|  | Bijendra Patel | United Kingdom |
|  | Alice Tsai | United Kingdom |
|  | Christoph Tschuor | Denmark |
|  | Gemma Vellalta | Spain |
|  | Ramon Villalonga | Spain |
| **Lower GI surgeons** | Manish Chand | United Kingdom |
|  | Audrius Dulskas | Lithuania |
|  | Antonio D’Urso | France |
|  | Nicola Eardley | United Kingdom |
|  | Eloy Espin-Basany | Spain |
|  | Giampaolo Formisano | Italy |
|  | Nikolaos Gouvas | Greece |
|  | Rosa Maria Jiménez-Rodríguez | Spain |
|  | Michail Klimovskij | United Kingdom |
|  | Gianluca Pellino | Italy |
|  | Thalia Petropoulou | Greece |
|  | Wanda Petz | Italy |
|  | Kapil Sahnan | United Kingdom |
|  | Theodosopoulos Theodosis | Greece |
| **GI surgical trainees** | Ademola Adeyeye | United Kingdom |
|  | Asma Afzal | United Kingdom |
|  | Daniel Campioni-Norman | United Kingdom |
|  | Bibek Das | United Kingdom |
|  | Michael Devine | United Kingdom |
|  | Lachlan Dick | United Kingdom |
|  | Jennifer Eckhoff | Germany |
|  | Dolores Krauss | Germany |
|  | Gita Lingam | United Kingdom |
|  | Pietro Mascagni | Italy |
|  | Tamsin Morrison | United Kingdom |
|  | Munir Tarazi | United Kingdom |
|  | Caoimhe Walsh | United Kingdom |
| **Robotic theatre team (anaesthetists, scrub nurses, robotic practitioners), psychologists and NOTSS experts** | Alexandra Aye Ivanuta | United Kingdom |
|  | Peter A Brennan | United Kingdom |
|  | Peter Buckle | United Kingdom |
|  | Michael Eichlseder | Germany |
|  | Samantha Evans | United Kingdom |
|  | Neli Harvey | United Kingdom |
|  | Ronalyn Hosena | United Kingdom |
|  | Hannah Kettley-Linsell | United Kingdom |
|  | Stella Mavroveli | United Kingdom |
|  | Freideriki Sifaki | Greece |
|  | Tayana Soukup | United Kingdom |
|  | Madeleine Wells | United Kingdom |
| **Robotic industry providers** | Lakshmi Balasubramanian | Intuitive Surgical |
|  | Niall Fahy | Medtronic |
|  | Harald Ginterstorfer | Intuitive Surgical |
|  | Elizabeth Jones | Medtronic |
|  | Sara Lazzaretti | Asensus Surgical |
|  | David Marante | Intuitive Surgical |
|  | Gregory de Martino | Intuitive Surgical |
|  | Anders Melander | Surgical Science |
|  | Shelley Petersen | Intuitive Surgical |
|  | James Scott | Intuitive Surgical |
|  | Mark Slack | CMR Surgical |
|  | Katherine Winger | CMR Surgical |
| **Patient representatives** | Julie Hepburn | United Kingdom |
|  | Pete Wheatstone | United Kingdom |

**Supplementary file 3.** Results of Delphi survey rounds one and two (critically important). ERAS, Enhanced Recovery After Surgery; CAT, Competency Assessment Tool; GEARS, Global Evaluative Assessment of Robotic Skills. * = new statement.

|  |  | **Round one -**  **critically important (7-9)** | **Round two -**  **critically important (7-9)** | **Achieved consensus for inclusion** | **Round achieved** |
| --- | --- | --- | --- | --- | --- |
| **Item** | **Section 1: Knowledge requirements of the GI robotic training curriculum** |  |  |  |  |
|  | **Key skills of a bedside assistant should include:** |  |  |  |  |
| 1  2  3  4  5  6  7  8  9 | *Preoperative:*  Knowledge of the theatre room set-up and patient position  Knowledge of the console and its functions  Knowledge of the different robotic systems  Knowledge of how to drape the robotic system  Knowledge of the physiology of pneumoperitoneum  Knowledge of procedure-specific port placement  Knowledge of patient specific characteristics (e.g. previous operations, tumour localisation)*  Knowledge of basic laparoscopic skills*  Knowledge of instrument-specific details for recharges (e.g. clips, staplers)* | 96%  62%  32%  69%  62%  81%  -  -  93% | -  63%  29%  70%  76%  -  80%  80%  - | Yes  No  No  Yes  Yes  Yes  Yes  Yes  Yes | 1  -  -  2  2  1  2  2  2 |
| 10  11  12  13  14  15  16  17  18  19  20  21  22  23 | *Intraoperative:*  Be able to perform safe entry of trocars, port placement and closure  Be able to dock the robotic system safely  Be able to troubleshoot and re-dock the robotic system  Be able to insert, change and remove robotic instruments  Be able to maintain a clear image  Be able to appropriately use the assistant port  Be able to demonstrate master manipulator control use of the foot pedal and hand clutches  Be able to demonstrate multi-arm control of the robotic instruments  Be able to demonstrate correct techniques for tissue handling and dissection  Be able to demonstrate use of stapler/advanced energy use  Be able to manage intraoperative complications (e.g. bleeding)  Be able to convert to laparoscopic/open surgery  Be able to perform planned and emergency undocking  Be able to manage collisions/arm clashes* | 85%  96%  96%  96%  83%  96%  45%  41%  54%  59%  75%  78%  96%  - | -  -  -  -  -  -  40%  44%  61%  83%  -  -  -  96% | Yes  Yes  Yes  Yes  Yes  Yes  No  No  No  Yes  Yes  Yes  Yes  Yes | 1  1  1  1  1  1  -  -  -  2  1  1  1  2 |
| 24  25  26  27  28 | *Non-technical skills:*  Be able to demonstrate situational awareness  Be able to communicate effectively with the team  Be able to demonstrate leadership skills  Be able to challenge all members of the team without fear if there are any safety concerns*  Be able to supervise/assist scrub practitioners in the training* | 97%  97%  69%  -  - | -  -  51%  77%  66% | Yes  Yes  No  Yes  No | 1  1  -  2  - |
|  | **Key skills of a console surgeon should include:** |  |  |  |  |
| 29  30  31  32  33  34  35  36 | *Preoperative:*  Knowledge of the theatre room set-up and patient position  Knowledge of the console and its functions  Knowledge of the different robotic systems  Knowledge of how to drape the robotic system  Knowledge of the physiology of pneumoperitoneum  Knowledge of procedure-specific port placement  Knowledge of the costs of the robotic consoles and use/reuse*  Knowledge of both bedside and console approach* | 94%  97%  58%  66%  89%  97%  -  - | -  -  44%  60%  -  -  66%  94% | Yes  Yes  No  No  Yes  Yes  No  Yes | 1  1  -  -  1  1  -  2 |
| 37  38  39  40  41  42  43  44  45  46  47  48 | *Intraoperative:*  Be able to perform safe entry of trocars, port placement and closure  Be able to dock the robotic system safely  Be able to troubleshoot and re-dock the robotic system  Be able to insert, change and remove robotic instruments  Be able to maintain a clear image  Be able to appropriately use the assistant port  Be able to demonstrate master manipulator control use of the foot pedal and hand clutches  Be able to demonstrate multi-arm control of the robotic instruments  Be able to demonstrate correct techniques for tissue handling and dissection, and suturing  Be able to manage intraoperative complications (e.g. bleeding)  Be able to convert to laparoscopic/open surgery  Be able to perform planned and emergency undocking | 93%  90%  90%  90%  96%  85%  99%  96%  97%  97%  97%  97% | -  -  -  -  -  -  -  -  -  -  -  - | Yes  Yes  Yes  Yes  Yes  Yes  Yes  Yes  Yes  Yes  Yes  Yes | 1  1  1  1  1  1  1  1  1  1  1  1 |
| 49  50  51  52  53  54  55 | *Non-technical skills:*  Be able to demonstrate situational awareness  Be able to communicate effectively with the team  Be able to demonstrate leadership skills  Be able to demonstrate ergonomic awareness and fatigue management*  Be able to demonstrate time management and pacing*  Be able to demonstrate to stress and crisis management*  Be able to demonstrate interdisciplinary collaboration* | 99%  99%  96%  -  -  -  - | -  -  -  80%  80%  94%  84% | Yes  Yes  Yes  Yes  Yes  Yes  Yes | 1  1  1  2  2  2  2 |

|  |  | **Round one -**  **critically important (7-9)** | **Round two -**  **critically important (7-9)** | **Achieved consensus for inclusion** | **Round achieved** |
| --- | --- | --- | --- | --- | --- |
| **Item** | **Section 2: Training components of the GI robotic curriculum** |  |  |  |  |
|  | **Key components of a robotic training curriculum for GI surgical trainees should include:** |  |  |  |  |
| 56  57  58  59  60  61  62  63  64  65  66 | E-learning modules  Core skills training (patient and port positioning, docking)  Virtual reality simulation training  Dual console training  Dry lab (model simulation)  Wet lab (animal simulation)  Wet lab (cadaveric simulation)  Bedside assisting  Live case observation (observation of robotic operations by an expert robotic surgeon)  Non-technical skills training  Emergency undocking and conversion training* | 82%  97%  83%  83%  96%  66%  69%  89%  94%  79%  - | -  -  -  -  -  70%  71%  -  -  -  96% | Yes  Yes  Yes  Yes  Yes  Yes  Yes  Yes  Yes  Yes  Yes | 1  1  1  1  1  2  2  1  1  1  2 |
|  | **Robotic e-learning modules for GI surgical trainees should include:** |  |  |  |  |
| 67  68  69  70  71  72  73  74  75  76  77  78  79  80 | History and development of robotic surgery  Introduction to robotic systems  Patient selection and preparation  Port placement  Docking  Troubleshooting  Emergency management/conversion to open or laparoscopy  Information on ERAS (enhanced recovery after surgery)  Non-technical skills  Laparoscopic surgery principles*  How to set up the robotic console (including ergonomics)*  Case-specific learning*  Robotic training curriculum*  Error recognition and recovery* | 27%  66%  92%  97%  99%  100%  96%  52%  63%  -  -  -  -  - | 17%  61%  -  -  -  -  -  39%  67%  69%  93%  93%  -  - | No  No  Yes  Yes  Yes  Yes  Yes  No  No  No  Yes  Yes  -  - | -  -  1  1  1  1  1  -  -  -  2  2  -  - |
|  | **Wet and dry lab robotic simulation metrics for GI surgical trainees should capture:** |  |  |  |  |
| 81  82  83  84  85  86  87  88  89  90  91  92  93  94  95  96  97  98  99 | Time spent on any given task  Number of attempts  Completion of tasks  Task scores  Error rate (e.g. instrument clash, loss of instrument view)  Proficient camera control  Appropriate use of the third arm  Exchange of arms  Use of hand and foot controllers at the console  Economy of movement in tissue dissection and suturing  Safe energy application  Learning curve assessment (time spent for acquisition of a new skill)*  Correct use of console (e.g. arms resting on the arm rest)  Instrument force application during tissue handling  Haptic feedback utilisation (if applicable)  Error recovery time  Hand-eye coordination efficiency  Eye tracking for situation awareness  Teamwork and communication performance in team-based scenarios | 58%  63%  90%  96%  87%  90%  83%  93%  94%  86%  93%  -  -  -  -  -  -  -  - | 57%  61%  -  -  -  -  -  -  -  -  -  71%  71%  91%  67%  76%  90%  80%  79% | No  No  Yes  Yes  Yes  Yes  Yes  Yes  Yes  Yes  Yes  Yes  Yes  Yes  No  Yes  Yes  Yes  Yes | -  -  1  1  1  1  1  1  1  1  1  2  2  2  -  2  2  2  2 |
|  | **Which metrics should be used for e-learning modules assessment?** |  |  |  |  |
| 100 | Time spent (i.e. minimum time requirement)  Knowledge test results  Other  None | 13%  75%  11%  1% | -  -  -  - | No  Yes  No  No | -  1  -  - |
|  | **Should virtual reality simulation be:** |  |  |  |  |
| 101 | Time-based training (specific number of hours required)  Proficiency-based training (certain level of specific skills required)  Other | 7%  89%  4% | -  -  - | No  Yes  No | -  1  - |
|  | **If time-based training, how many hours are required for virtual reality sign-off before live console training?** |  |  |  |  |
| 102 | 1-10 hours  11-20 hours  21-30 hours  31-50 hours  50+ hours | 0%  0%  60%  0%  40% | -  -  -  -  - | No  No  No  No  No | -  -  -  -  - |
|  | **If proficiency-based training, which skills are required for virtual reality sign-off and which score before live console training** *(results for VR score ≥ 90% shown)?* |  |  |  |  |
| 103 | Endowrist manipulation  Camera control  Knot tying  Tissue dissection | 75%  79%  63%  76% | -  -  54%  - | Yes  Yes  No  Yes | 1  1  -  1 |
| 104 | **Dry and wet lab skills training requires which of the following models** *(results for high-fidelity models shown):* |  |  |  |  |
|  | Familiarisation with the robotic instruments and early stage of training  Use of energy and haemostasis  Advanced dissection skills | 68%  62%  55% | 70%  64%  47% | Yes  No  No | 2  -  - |

|  |  | **Round one -**  **critically important (7-9)** | **Round two -**  **critically important (7-9)** | **Achieved consensus for inclusion** | **Round achieved** |
| --- | --- | --- | --- | --- | --- |
| **Item** | **Section 3: Performance assessment of robotic training in GI surgery** |  |  |  |  |
|  | **Technical skills assessment for GI surgical trainees to progress to independent practice should include** |  |  |  |  |
| 105  106  107  108  109  110 | Case logs  Subjective proctored sign-off as competent without the implementation of objective assessment tools  Global Rating Scale (e.g. GEARS)  Procedure-specific tool (e.g. CAT)  Video assessment  Assessment over period of time including skill progress over subsequent procedures and increasing case complexity | 73%  85%  80%  83%  87%  90% | -  -  -  -  -  - | Yes  Yes  Yes  Yes  Yes  Yes | 1  1  1  1  1  2 |
|  | **When robotic console operating, clinical metrics for GI surgical trainees should capture** |  |  |  |  |
| 111  112  113  114  115  116  117  118  119  120  121  122 | Number of hours on the console  Near misses during operations  Minimum number of procedures performed  Complication rates (e.g. major haemorrhage)  Conversion rate to open or laparoscopy  Histopathological outcomes (if applicable)  Return to operating theatre  Readmission rates  Number of times on a dual console that the other surgeon had to take over*  Error recovery time*  Number of key steps of procedure completed*  Instrument movements (e.g. use of additional arm, movements of camera)* | 68%  86%  83%  89%  78%  73%  78%  72%  -  -  -  - | 74%  -  -  -  -  -  -  -  69%  81%  -  - | Yes  Yes  Yes  Yes  Yes  Yes  Yes  Yes  No  Yes  -  - | 2  1  1  1  1  1  1  1  -  2  -  - |
|  | **Operative video assessment for GI surgical trainees should primarily include:** |  |  |  |  |
| 123 | Key steps of operative video assessment by unblinded expert(s)  Key steps of operative video assessment by blinded expert(s)  Key steps of operative video assessment by blinded expert and proctor*  Full video assessment by unblinded expert(s)  Full video assessment by blinded expert(s)  Full video assessment by blinded expert and proctor*  Automation of video assessment using artificial intelligence*  No video assessment required | 7%  37%  -  11%  37%  -  -  1% | 33%  60%  73%  27%  23%  50%  49%  3% | No  No  Yes  No  No  No  No  No | -  -  2  -  -  -  -  - |
|  | **Should the trainee operative video be assessed by:** |  |  |  |  |
| 124 | One expert  Two experts  None | 24%  73%  3% | -  -  - | No  Yes  No | -  1  - |

|  |  | **Round one -**  **critically important (7-9)** | **Round two -**  **critically important (7-9)** | **Achieved consensus for inclusion** | **Round achieved** |
| --- | --- | --- | --- | --- | --- |
| **Item** | **Section 4: Requirements for certification and supervision** |  |  |  |  |
|  | **The following are required for safe robotic platform sign-off for GI surgical trainees** |  |  |  |  |
| 125  126  127  128  129  130  131  132  133  134  135 | Safe completion of robotic procedures with independent assessment by proctor  Independent evaluation of robotic videos  Assessment of robotic operations using objective assessment metrics  Assessment of robotic operations using clinical outcome metrics (i.e. intraoperative injury, conversion)  Assessment methods clearly demonstrating improved performance over time (i.e. learning curve)  Safe completion of a robotic training programme (defined pass/fail sign-off)  Completion of a robotic fellowship programme  Procedures should be demonstrated in basic cases first before advanced cases  Re-certification is required when moving to a different robotic system  Minimum number of bedside assisted cases for console surgeon*  Online examinations* | 93%  82%  87%  85%  89%  92%  63%  85%  78%  -  - | -  -  -  -  -  -  66%  -  -  86%  - | Yes  Yes  Yes  Yes  Yes  Yes  No  Yes  Yes  Yes  - | 1  1  1  1  1  2  2  1  1  1  - |
|  | **How important are the following for assessing the long-term impact or success of the GI robotic training curriculum per individual** |  |  |  |  |
| 136  137  138  139  140  141  142 | Revalidation  Number of procedures per year  Clinical outcomes e.g. percentage of complications per year  Stepwise certification (i.e. Level 1, 2, 3 followed by diploma)*  Number of trainees trained by individual*  Increase in the number of assisted and performed cases, and a reduction in the number of observed cases*  Non-technical skills e.g. team communication effectiveness tracking, situation awareness assessments, stress and impact fatigue studies* | 65%  80%  86%  -  -  -  - | 66%  -  -  74%  50%  81%  74% | No  Yes  Yes  Yes  No  Yes  Yes | -  1  1  2  -  2  2 |
|  | **Who should primarily sign-off trainees to say they are practically safe and independent?** |  |  |  |  |
| 143 | Trainee self-directed  Trainer/Mentor  Societal/board certification  Regional training body  National training body  Industry provider  External independent assessor*  Other | 1%  42%  28%  6%  13%  0%  -  10% | 4%  81%  57%  23%  47%  10%  34%  1% | No  Yes  No  No  No  No  No  No | -  2  -  -  -  -  -  - |

**Supplementary file 4.** Microsoft Excel document of ERSC robotic training curriculum.
